# Neuronal Ndrg4 Is Essential for Nodes of Ranvier Organization in Zebrafish

**DOI:** 10.1371/journal.pgen.1006459

**Published:** 2016-11-30

**Authors:** Laura Fontenas, Flavia De Santis, Vincenzo Di Donato, Cindy Degerny, Béatrice Chambraud, Filippo Del Bene, Marcel Tawk

**Affiliations:** 1 U1195, Inserm, University Paris Sud, University Paris-Saclay, Kremlin-Bicêtre, France; 2 Institut Curie, PSL Research University, Paris, France; Centre for Neuroregeneration, Edinburgh, UNITED KINGDOM

## Abstract

Axon ensheathment by specialized glial cells is an important process for fast propagation of action potentials. The rapid electrical conduction along myelinated axons is mainly due to its saltatory nature characterized by the accumulation of ion channels at the nodes of Ranvier. However, how these ion channels are transported and anchored along axons is not fully understood. We have identified N-myc downstream-regulated gene 4, ndrg4, as a novel factor that regulates sodium channel clustering in zebrafish. Analysis of chimeric larvae indicates that ndrg4 functions autonomously within neurons for sodium channel clustering at the nodes. Molecular analysis of ndrg4 mutants shows that expression of snap25 and nsf are sharply decreased, revealing a role of ndrg4 in controlling vesicle exocytosis. This uncovers a previously unknown function of ndrg4 in regulating vesicle docking and nodes of Ranvier organization, at least through its ability to finely tune the expression of the t-SNARE/NSF machinery.

## Author Summary

Myelination is an important process that enables fast propagation of action potential along the axons. Schwann cells (SCs) are the specialized glial cells that ensure the ensheathment of the corresponding axons in the Peripheral Nervous System. In order to do so, SCs and axons need to communicate to organize the myelinating segments and the clustering of sodium channels at the nodes of Ranvier. We have investigated the early events of myelination in the zebrafish embryo. We here identify ndrg4 as a novel neuronal factor essential for sodium channel clustering at the nodes. Immuno-labeling analysis show defective vesicle patterning along the axons of ndrg4 mutants, while timelapse experiments monitoring the presence and the transport of these vesicles reveal a normal behavior. Molecular analysis unravels a novel function of ndrg4 in controlling the expression of the t-SNARE/NSF machinery required for vesicle docking and release. However, inhibiting specifically regulated synaptic vesicle release does not lead to sodium channel clustering defects. We thus propose that ndrg4 can regulate this process, at least partially, through its ability to regulate the expression of key components of the t-SNARE/NSF machinery, responsible for clustering of sodium channels along myelinated axons.

## Introduction

Myelination is a vertebrate adaptation that ensures the fast propagation of action potentials along the axons. Schwann Cells (SCs) are one of the myelinating glial cells of the Peripheral Nervous System (PNS) while Oligodendrocytes (OLs) are responsible for myelin wrapping in the Central Nervous System (CNS) [1–7]. While myelin sheaths insulate axons and inhibit current leakage, nodes of Ranvier found at regular intervals, gather voltage-gated sodium channels in clusters, and therefore are the only places where action potentials are regenerated, allowing their rapid propagation along axons [8–10]. Defective myelin sheaths or nodes of Ranvier prevent the efficient conduction of action potentials and severely impairs axonal function. Several signaling pathways, mainly intrinsic to SCs, have been identified as being positive or negative regulators of peripheral myelination [11–14]. Analysis of zebrafish mutants lacking SCs (e.g. *erbb2*, *erbb3*, *sox10/cls*) shows defects in sodium channel clustering and positioning [15], suggesting that SCs give essential instructive cues for the proper organization of myelinated axons. However, less is known about neuronal factors that ensure a proper myelin organization so that ion channels are mainly concentrated at the repetitive nodes of Ranvier along myelinated axons.

To better understand the molecular mechanisms governing peripheral myelination, and since the formation of the nodes depends on the interaction between neurons and glia, we undertook a differential screen to look for genes that are dysregulated in the absence of SCs in zebrafish. We compared the transcriptomes of the GFP+ and GFP- cells in the foxd3::GFP transgenic line (through FACS sorting), in groups of embryos that contain or not Schwann cells (following a sox10 knockdown). We have identified a neuronal factor, ndrg4, as a major regulator of sodium channel clustering at the nodes of Ranvier.

Ndrg4 belongs to the NDRG (N-myc Downstream-Regulated Gene) family, which includes four related members, known to be important in tumorigenesis and linked to a range of cancers [16,17]. The function of Ndrg4 itself has been extensively studied in cancer although conflicting results showed that Ndrg4 has either a tumor-suppressive or an oncogenic function depending on the tissue [17]. NDRG1 is the most widely studied protein, namely for its role in peripheral myelination since a mutation in this gene leads to a severe autosomal recessive demyelinating neuropathy, NDRG1-linked Charcot-Marie-Tooth Disease (CMT4D) [18–20]. While NDRG1 function in myelination is well established, the role of NDRG4 in this process is still unknown. The latter is mainly expressed in the nervous system and the heart of mice and zebrafish [21]. In the mouse embryo, an indirect role of NDRG4 in severe ventricular hypoplasia has been proposed [22] while in zebrafish, Ndrg4 is required for normal myocyte proliferation during early cardiac development [21]. Given its expression in the brain, it has been suggested that NDRG4 might play an important role within the CNS. Indeed, the expression of brain-derived neurotrophic factor (BDNF) is reduced in the cortex of Ndrg4 KO mice that leads to spatial learning and memory defects [23]. A possible role of NDRG4 in neuronal differentiation and neurite formation has also been proposed following *Ndrg4* manipulation in PC12 cells [24]. Finally, a significant decrease in NDRG4 expression has been reported in Alzheimer disease brains [25].

Here, we identify a novel function for zebrafish ndrg4, in controlling vesicle fusion and release by regulating, among others, the levels of the t-SNARE protein, Snap25 (Synaptosomal Associated Protein 25KDa), known to be required for the docking and merging of vesicles with the cell membrane during exocytosis [26,27]. Thus, in addition to their pronounced heart defects, the zebrafish ndrg4 mutants are paralyzed. Our results reveal a previously unknown neuronal role for ndrg4 in sodium channel clustering that is most likely due to its ability to regulate the expression of key components of the t-SNARE/NSF machinery.

## Results

### Sodium channel clustering is dependent on ndrg4 function

**Fig 1:**
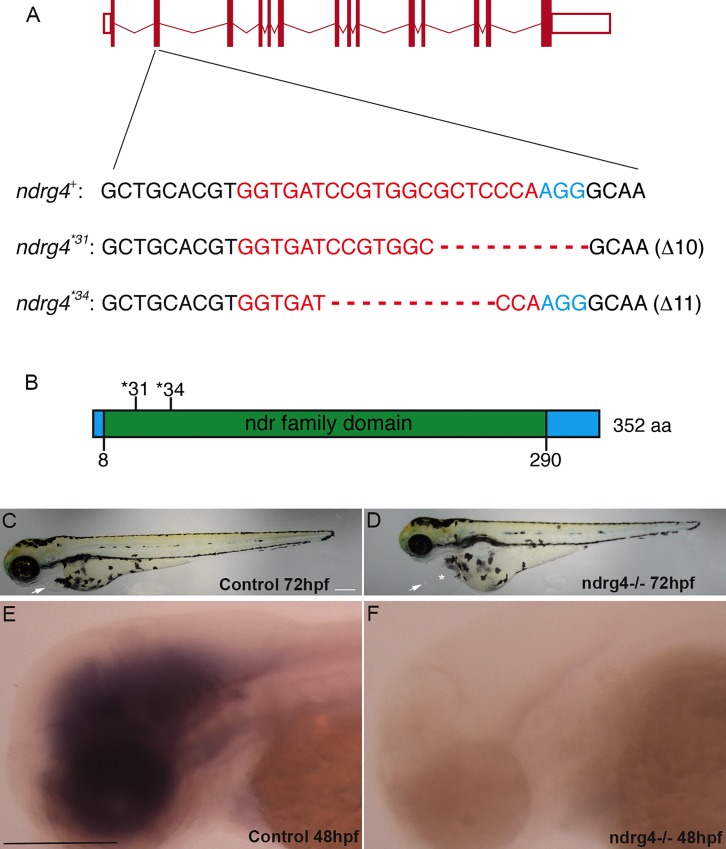
Characterization of the ndrg4 mutant. (A) Schematic representation of the *ndrg4* genomic locus. The extended region on the *exon 2* represents the sequence targeted by the CRISPR/Cas9 system. Red: sgRNA binding site. Blue: PAM sequence. *ndrg4*^*+*^ corresponds to the wild-type allele; *ndrg4* *^31^ and *ndrg4* *^34^ are the loss-of-function alleles used in this study. (B) Schematic of ndrg4 protein product. In *ndrg4* *^31^ and *ndrg4* *^34^ mutant fish, the deletions result in a frameshift generating a premature STOP codon at the level of the amino acids 31 and 34 (of 352) in the ndr family domain. Lateral views of a control (C) and a ndrg4 mutant (D) embryos at 72 hpf. The arrows point to the heart, note the pronounced heart edema (white asterisk) observed in the ndrg4 mutant. Lateral view of *ndrg4* mRNA expression in a control (E) and a ndrg4-/- embryo (F) at 48hpf. Note the absence of *ndrg4* expression in the mutant. Scale bar = 200 μm.

**Fig 2:**
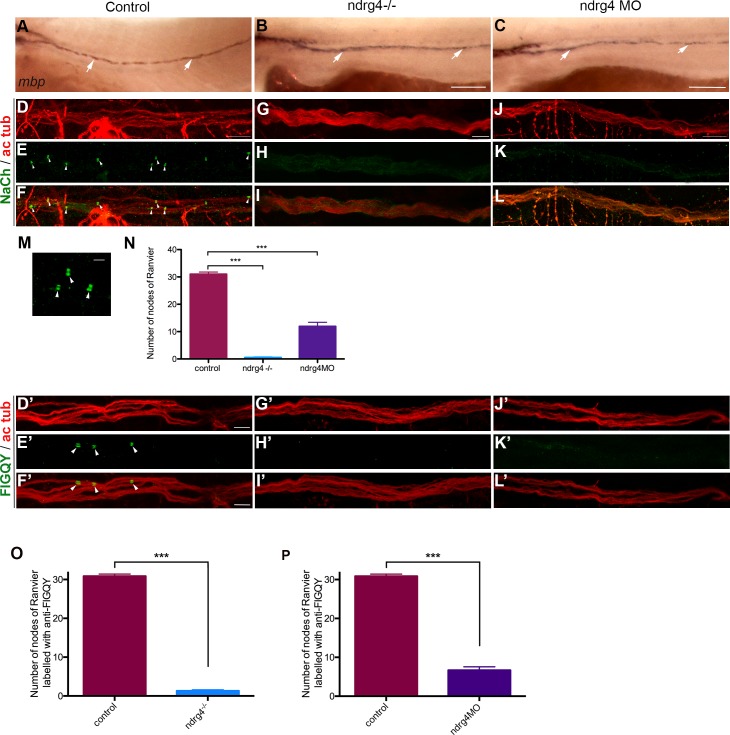
ndrg4 is required for sodium channel and neurofascin clustering in the peripheral nervous system. (A-C) Lateral view of *mbp* RNA expression in control (A), ndrg4 mutant (B) and morphant (C) embryos at 4 dpf. Arrows indicate *mbp*-expressing cells along the PLLn. Scale bars = 200μm. (D-L) Acetylated tubulin (ac tub; red) and sodium channels (NaCh; green) immunohistochemistry of a (D-F) control, ndrg4 mutant (G-I) and ndrg4 morphant (J-L) PLLn at 4 dpf. Scale bars = 5μm. (M) High magnification of three nodes of Ranvier (arrowheads) from a control nerve. Scale bar = 100nm. (N) A significant decrease in the number of sodium channels clusters is observed in ndrg4 mutants and morphants in comparison to controls (p<0.001). Acetylated tubulin (ac tub; red) and FIGQY (green) immunohistochemistry of a (D’-F’) control, ndrg4 mutant (G’-I’) and ndrg4 morphant (J’-L’) PLLn at 4dpf. Scale bars = 5 μm. (O,P) A significant decrease in the number of FIGQY labeled clusters is observed in ndrg4 mutants and morphants in comparison to controls (p<0.001).

Having identified a dysregulation in the expression of ndrg4 in a differential screen of normal and SCs deficient embryos, we wanted to assess its function during PNS myelination, thus, we generated a ndrg4 mutant using CRISPR/Cas9 technology [28]. The introduced mutation begets a deletion in the ndrg4 DNA sequence and introduces a premature stop codon in the *ndrg4* mRNA sequence leading to a nonsense-mediated decay of the corresponding mRNA transcript (Fig 1A, 1B, 1E and 1F). A concomitant knockdown approach using a specific ndrg4 splice blocking Morpholino (MO; 0.6 pmole/embryo) and a control 5 base pair mismatch ndrg4 MO (0.6 pmole/embryo) was simultaneously used during this study ([21] and S1 Fig). A pronounced heart edema and a complete paralysis of the embryos were the first obvious defects observed in these mutants and morphants starting from 48 hours post fertilization (hpf) (the earliest time point analyzed here) (Fig 1C and 1D; S1 Fig). Ndrg4 homozygous mutants and morphants failed to respond to touch test at 3 days post fertilization (dpf) (S1, S2 and S3 Movies). The embryos looked thinner and shorter in comparison to controls and had slightly smaller eyes (Fig 1C and 1D; S1 Fig). We first observed, using *in situ* hybridization, that the majority of ndrg4 mutants (30 out of 38 embryos) showed no obvious change in the expression of *myelin basic protein* (*mbp*) at 4dpf (Fig 2A–2C), a major protein of the myelin sheath and commonly used marker of myelination, compared to control embryos (75 out of 80 embryos). This result suggests that ndrg4 function is not required *per se* for *mbp* expression.

We next investigated sodium channel distribution and organization along the PLLn. Using whole mount immunohistochemistry for voltage-gated sodium channels (anti-panNa_v_Ch) and axons (anti-acetylated tubulin) at 4 dpf, we visualized many sodium channels concentrated in clusters at the nodes of Ranvier within the control PLLn (Fig 2D–2F and 2M). However, in ndrg4 mutants and morphants, we noticed that sodium channels were not clustered at the nodes of Ranvier (Fig 2G–2I and 2J–2L). We quantified the number of nodes of Ranvier within the PLLn in the last 8 somites starting from the most posterior neuromast of the larvae. We counted an average of 31± 2.49 nodes of Ranvier in control axons (n = 13 embryos), whereas we found only 0.5± 0.8 nodes in the ndrg4 mutants (n = 14 embryos) and 11±5.20 nodes in ndrg4 morphants (n = 13 embryos) (Fig 2N). We have also looked at the clustering of sodium channels in the sox10:mRFP transgenic line that labels membrane extensions of SCs [29]. We could see clusters of sodium channels localized in the gaps between adjacent internodal segments in controls at 4dpf (average of 4.42± 1.08 clusters per somite; 4 different embryos) (S1G Fig), while fewer clusters were observed in these gaps in ndrg4 morphants (average of 1.90± 0.84 clusters per somite; 5 different embryos; p<0.0001) (S1G Fig). This result suggests that ndrg4 function is required for sodium channel clustering along the axons.

We next labeled embryos with antibodies against a sequence (FIGQY) conserved in neurofascin family of adhesion molecules, and recognize the neurofascin 186. This latter is also localized at nodes of Ranvier in mammals and zebrafish, and shown to co-localize with NaCh clusters in zebrafish larvae [10,30–33]. Similar results were observed; we noticed that the FIGQY antigen labeling was diffused in ndrg4 mutants and morphants (Fig 2G’–2L’), in comparison to the clustered labeling observed in controls (Fig 2D’–2F’). We counted an average of 30.86 ±0.54 clusters in the last 8 somites within the PLLn in control embryos (n = 14 embryos) whereas we found only 1.29 ±0.29 in ndrg4 mutants (n = 14 embryos) and 6.69 ±0.86 in ndrg4 morphants (n = 13 embryos) (Fig 2O and 2P). This result suggests that initial clustering of neurofascin at the nodes is also dependent on ndrg4 function.

### Ndrg4 is not required for PLLn growth or early SCs development

**Fig 3:**
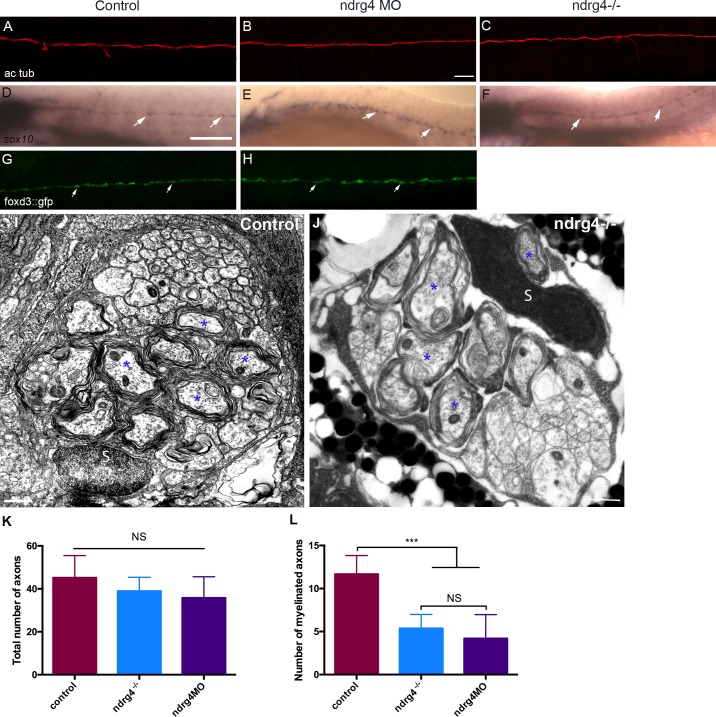
ndrg4 is not required for axonal outgrowth or early Schwann cell development. Acetylated tubulin expression in control (A), ndrg4 mutant (C) and morphant (B) embryos at 4 dpf showing the PLLn nerve. Scale bar = 45μm. (d-F) Whole mount *in situ* hybridization of a (d) control embryo, ndrg4 mutant (F) and ndrg4 morphant (E) showing *sox10* expression in PLLn SCs (arrows) at 3 dpf. Scale bar = 200μm. Lateral view of a control foxd3::GFP embryo (G), a ndrg4 morphant (H) at 3 dpf showing SCs (arrows) along the PLLn. Transmission electron micrographs showing cross-section through (I) control and ndrg4 mutant (J). Control PLLn shows an average of 10.7 myelinated axons (blue asterisks). (J) An average of 5.36 myelinated axons (blue asterisks) is observed in the ndrg4 mutant’s PLLn. (S: Schwann cell). Scale bars = 0.5μm. (K,L) Quantification of the total number of axons and the number myelinated axons in controls, ndrg4 mutants and ndrg4 morphants. NS: Non Significant.

To test whether SC and axonal development occurs normally in ndrg4 mutants and morphants, we first performed a whole mount acetylated tubulin immunostaining for axonal labeling. We observed no significant difference in PLLn axonal outgrowth (Fig 3A–3C) between mutants (n = 14 embryos), morphants (n = 18 embryos) and controls (n = 20 embryos) at 4 dpf, indicating that axonal growth and maintenance are not defective in ndrg4 mutants. We then examined *sox10* mRNA expression at 72 hpf. Sox10 is a transcription factor that labels neural crest cells including SC progenitors[34–36]. Ndrg4 mutants (n = 11 embryos) (Fig 3F) and morphants (n = 12 embryos) (Fig 3E) were comparable to controls (n = 32 embryos) (Fig 3D), showing a similar expression of *sox10* along the PLLn, confirming the normal development and distribution of SCs. We also took advantage of the foxd3::GFP larvae which express the Green Fluorescent Protein (GFP) in SCs [37] to look for SCs migration in ndrg4 morphants. We observed no significant difference in SC migration and maintenance between morphants (n = 20 embryos) and controls (n = 16 embryos) at 3 dpf (Fig 3G and 3H). Moreover, since the NDRG proteins are known to play a significant role in cancer, we assessed SC proliferation throughout their development. For this purpose, we performed an anti-phosphorylated histone 3 (PH3) labeling in foxd3::gfp larvae at 30, 48 and 72 hpf. Quantification of PH3 positive SCs did not show any significant difference between controls (10 embryos at 30hpf, 23 at 48hpf and 12 at 72hpf) (S2A Fig) and morphants (7 embryos at 30hpf, 11 at 48hpf and 10 at 72hpf) (S2B Fig). The quantification of this phenotype showed that the rate of SC proliferation was not significantly different in ndrg4 morphant embryos throughout development (S2C Fig). These data suggest that ndrg4 function is not required for early SC development and axonal growth. To further investigate later aspects of axonal development and SC myelination, we analyzed the ultrastructure of axons in the PLLn using Transmitted Electron Microscopy (TEM). The total number of axons in ndrg4 mutants and morphants was slightly but not significantly decreased in comparison to controls; we counted an average of 43.6 ±2.69 axons in controls (n = 10 nerves from 9 different embryos) at 4dpf, 38.93 ±1.66 axons in ndrg4 mutants (n = 11 nerves from 7 different embryos) and 35.8 ±4.4 axons in ndrg4 morphants (n = 5 nerves from 3 different embryos) (Fig 3I–3K). However, the number of myelinated axons was significantly reduced in ndrg4 mutants and morphants in comparison to controls, we could count an average of 5.36 ±0.49 myelinated axons in ndrg4 mutants and 4.2 ±1.24 in ndrg4 morphants in comparison to an average of 10.7 ±0.68 myelinated axons in controls (Fig 3L). This result suggests that ndrg4 function may, directly or indirectly, amend SC ability to myelinate but it is not essential for SC myelination as seen for sodium channel clustering.

### Ndrg4 is expressed in the Posterior Lateral Line ganglion (PLLg) but not in SCs

Overall, our results strongly suggest that ndrg4 function is required for nodes of Ranvier organization. To investigate whether its function is neuronal or intrinsic to SCs, we first looked at *ndrg4* expression. Like mammalian *Ndrg4* [20,23], zebrafish *ndrg4* is mainly expressed in the developing nervous system and heart [21]. Using *in situ* hybridization, we here confirm *ndrg4* expression within the brain, the eyes and the PLLg at 30 hpf (S3 Fig). The expression of *ndrg4* persists in the nervous system and specifically in the PLLg until at least 72 hpf (S3 Fig). However, *ndrg4* was not observed in SCs at any of these time points. This indicates that, at least in the zebrafish PNS, *ndrg4* is expressed in neuronal cells and not in glia.

### Ndrg4 function is required in neurons for sodium channel clustering

**Fig 4:**
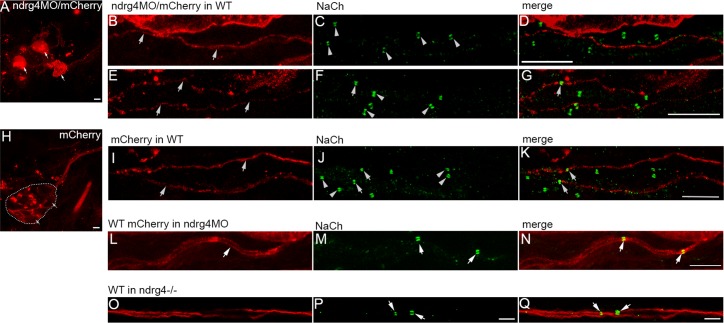
Chimeric embryos show evidence of ndrg4 requirement in neurons for sodium channel clustering. (A) ndrg4MO mCherry labeled PLL neurons shown by arrows. (b,e) ndrg4MO mCherry labeled axons of the PLLn in two different chimeric embryos; (c,f) sodium channels along the PLLn of ndrg4MO mCherry labeled (arrow) and of control (arrowheads) axons; (d,g) merge of the two labelings. Sodium channel clustering is absent in ndrg4MO axons (mCherry labeled) while control ones in the same PLLn show normal clustering. (H) Control mCherry labeled neurons indicated by arrows. The dashed line indicates the margin of the PLLg. (I) Control mCherry labeled axons (arrows). (J) Sodium channel clusters along the PLLn in control labeled (arrows) and other non-labeled (arrowheads) axons. (K) Merge of the two labelings. For (a, H) scale bars = 5μm. For (b-G; I-K) scale bars = 10μm. (L-N) WT mCherry labeled axons in ndrg4 morphant embryos are shown in (L, arrow) and the corresponding sodium channels in (M, arrows). Note the clustering of the nodes in the WT labeled axons while the other ndrg4 deficient axons show no sign of sodium channel clustering. (N) Merge of the two labelings. Scale bar = 7 μm. (O-Q) WT mcherry labeled axons in ndrg4-/- are shown in (O) and the corresponding sodium channels in (P, arrows). (Q) Merge of the two labelings showing clustered sodium channels along the WT axons (arrows). Scale bar = 5 μm.

SC activity and axon-SCs interaction are both required for clustering of sodium channels at the nodes of Ranvier [10,15]. Therefore, we asked whether ndrg4 function is required in neurons or in SCs despite a clear *ndrg4* mRNA expression in neurons and not in the glia of the zebrafish PNS (present data and [21]). A similar distribution profile was also observed for NDRG4 protein in the mouse CNS [20,23]. We therefore chose to specifically manipulate ndrg4 function in the PLLg. In order to do so, we first generated mosaic PLLg of WT and ndrg4 morphant cells by introducing ndrg4 morphant cells, co-injected with *mCherry* mRNA, into a WT background. In such chimeras, no or very few sodium channel clustering (0.05 ±0.23 cluster per somite) was observed along the PLLn axons derived from ndrg4 morphant PLLg neurons (19 somites from 4 different embryos) (Fig 4A–4G) in comparison to control PLLg cells (14 somites from 4 different embryos) where sodium channel clustering was always observed (2.1±1.8 clusters per somite, p<0.001) (Fig 4H–4K). We then introduced WT cells, labeled with *mCherry*, into a ndrg4 morphant background whereby SCs are defective for ndrg4 but the introduced PLLg neurons express normal levels of *ndrg4*. In this case, we can observe normal sodium channel clustering along these axons (2.6±1.2 clusters per somite) (Fig 4L–4N; 3 different embryos) while surrounding axons show little or no sign of sodium clustering. The same result was obtained when introducing WT cells into a ndrg4-/- background, where normal sodium channel clustering was observed along the WT axons (2.2±1.3 clusters per somite) (Fig 4O–4Q; 2 different chimeras). This result indicates that ndrg4 function is required cell autonomously in neurons for sodium channel clustering.

### Ndrg4 function is required for the expression of key genes essential for vesicle docking

To understand the molecular mechanisms governing neuronal ndrg4 function that leads to such defects, we undertook a differential microarray analysis looking for downstream targets that are dysregulated at 3dpf following ndrg4 knockdown. Total RNAs were extracted and compared between two groups of either 1.control embryos or 2.ndrg4 morphants (see Materials and Methods). In addition to a significant decrease in the expression of a number of genes known to be involved in hematopoiesis, related to ndrg4 expression and function in the heart, e.g. alas2 (Fold change (FC) 41, [38]); klfd (FC 17, [39]), that we will not discuss here, one particular major group of genes related to ndrg4 function in the nervous system was discerned. It appeared that ndrg4 significantly modulates the expression of numerous genes involved in vesicular release (e.g. caly, syt1a, snap25, nsf) and synaptic activity (e.g. syn2, rims2, sypa) (S1 Table). These data pointed to a previously unknown role for ndrg4 in regulating the expression of several key genes required for vesicle docking and fusion during exocytosis and synaptic activity.

**Fig 5:**
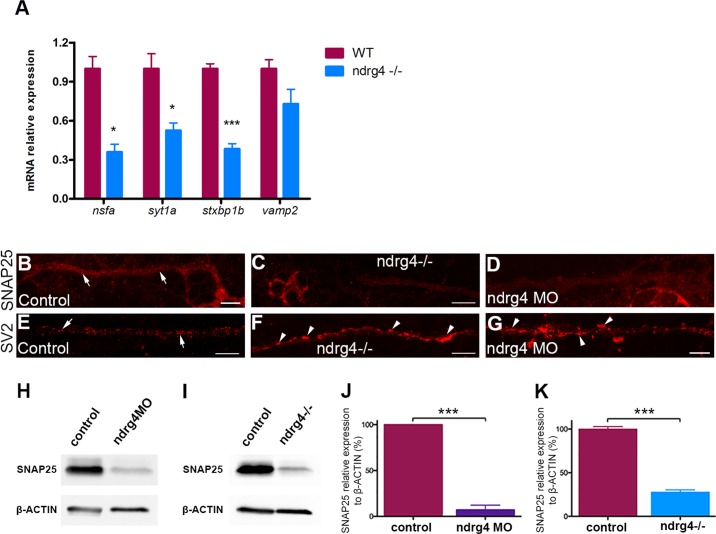
ndrg4 function is required for the expression of several genes that are essential for vesicle docking. (A) qPCR showing a significant decrease in the expression of nsfa, syt1a, stxbp1b but not vamp2 in ndrg4 mutants with mRNA relative expression to ef1a. Controls from 3 different experiments have deliberately been set to 100 per cent. (n = 3 independent experiments of 30 embryos each). (B-D) Lateral views showing Snap25 expression in the PLLn axons at 4 dpf. Snap25 is visible all along the PLLn (arrows) in controls (B), but detected to a lesser extent in the ndrg4 mutants (C) and morphants (D) PLLn. (E-G) Lateral views of SV2 immunostaining, labeling synaptic vesicles at 4 dpf. (E) Synaptic vesicles are regularly distributed in the control PLLn (arrows) whereas they form agglomerates (arrowheads) in ndrg4 mutants (F) and morphants (G). Scale bars = 10μm. (H-K) Western blots showing a sharp decrease in Snap25 protein expression in ndrg4 mutants and morphants. Snap25 expression is significantly decreased in ndrg4 morphants (H,J) and mutants (I,K) with protein relative expression to β-actin. Controls from 3 different experiments have deliberately been set to 100 per cent. (n = 3 independent experiments of 30 embryos each). For the mutants study, the average of three groups of controls has been set to 100 per cent.

To further confirm these results in ndrg4 mutants, we performed quantitative PCR (qPCR), western blots and whole mount immunochemistry experiments to look for specific changes in the expression of the main corresponding genes and proteins. Indeed, we observed a 65, 48 and 62 per cent decrease in the expression of n-ethylmaleimide sensitive factor a (nsfa), synaptotagmin1a (syt1a) and syntaxin binding protein1b (stxbp1b) respectively in ndrg4 mutants in comparison to controls (Fig 5A). However, the expression of the v-SNARE vamp2 (synaptobrevin), that is localized to vesicles and not to target membranes, was not altered (Fig 5A). Moreover, we could detect a 72 per cent decrease in the expression of Snap25 protein in ndrg4 mutants in comparison to controls (Fig 5I and 5K) at 3dpf. Similarly, ndrg4 knockdown led to a 90 per cent decrease in Snap25 protein expression (Fig 5H and 5J) (n = 3 independent experiments, p<0.001) in comparison to controls, showing a very sharp decrease in the expression of this key protein involved in vesicle docking and release. We next looked for Snap25 protein expression specifically in the PLLg and PLLn using whole mount immunochemistry at 4 dpf. We could observe a significant decrease in the expression of Snap25 along the PLLn and PLLg of ndrg4 mutants and morphants (Fig 5C and 5D, S1I and S1K Fig) compared to controls (Fig 5B, S1H and S1K Fig).

This result validates the overall decrease in the expression of different components essential for vesicle docking and release and for synaptic activity in ndrg4 mutants. Moreover, it shows the decrease in Snap25 expression in the PLLn and PLLg of ndrg4 mutants and morphants.

### Vesicle patterning is defective in ndrg4 mutants but the formation of vesicles and their transport are normal

Since the expression of several main components required for vesicular docking and release is significantly affected in ndrg4 mutants, we took a closer look at the distribution of vesicles along the PLLn at 4dpf using an anti-SV2 antibody. While synaptic vesicles showed a regular dotted pattern along the nerve of control embryos (n = 22 embryos) (Fig 5E), we could observe irregular agglomerates (Fig 5F and 5G) in ndrg4 mutants (n = 14 embryos) and morphants (n = 10 embryos), suggesting a defect in their release but not their formation.

It has been shown that the clustering of the channels at the nodes relies on vesicular axonal transport [40,41]. Thus, to test a possible role of ndrg4 in longitudinal vesicular trafficking, that might explain the lack of sodium channel clustering along the axons, we monitored mitochondrial and vesicular movements along the axons using time-lapse imaging. For this purpose, we injected *mito*::*GFP* and *rab5*::*YFP* mRNAs at one cell stage and the PLL nerve vesicular trafficking was analyzed at 48 hpf. Mitochondrial average velocity was comparable between controls (S4 Movie; 0.73 ±0.09μm.s^-1^; 56 mitochondria from 5 embryos) and ndrg4 morphants (S5 Movie; 0.76 ±0.2μm.s^-1^; 79 mitochondria from 5 embryos). Rab5 vesicles average velocity was rather slightly but not significantly increased in ndrg4 morphants (S6 Movie; 1.82 ±0.55μm.s^-1^; 91 vesicles from 5 embryos) in comparison to controls (S7 Movie; 1.31 ±0.44μm.s^-1^; 100 vesicles from 5 embryos). Altogether, these results suggest that ndrg4 is not required, *per se*, for vesicular formation or longitudinal transport along the axons.

### Snap25 knockdown leads to a decrease in the clustering of sodium channels along the PLLn

**Fig 6:**
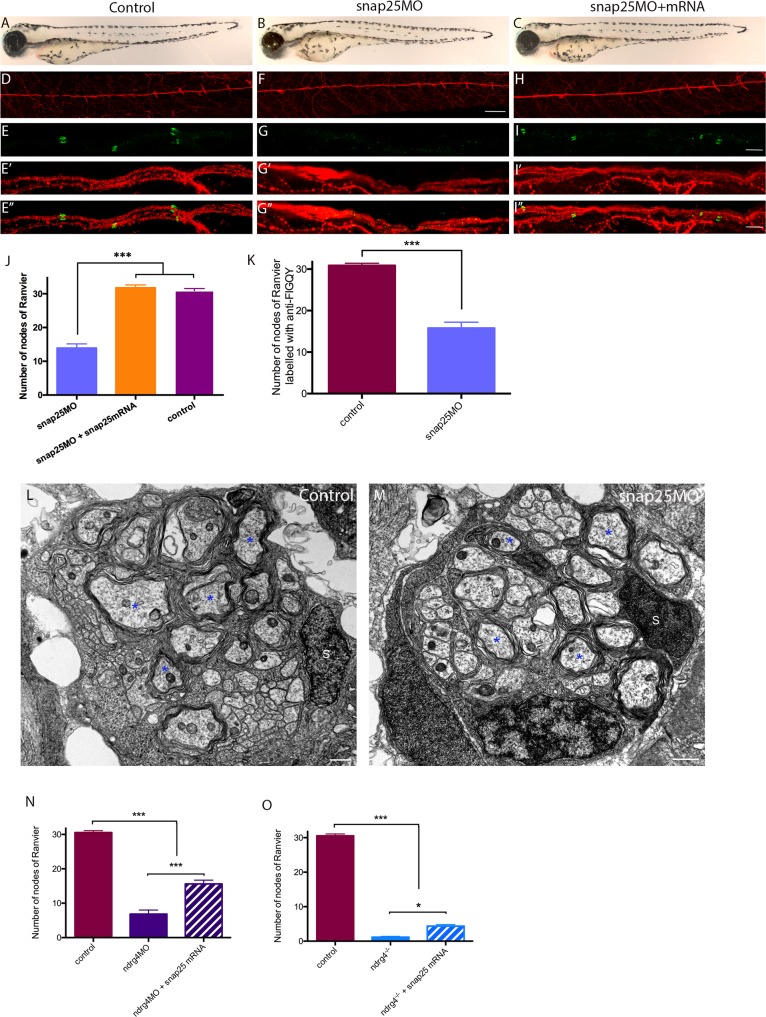
snap25 can regulate the clustering of sodium channels and neurofascin along the PLLn and increase the clustering of sodium channels in ndrg4 mutants. (A-C) lateral views of control (A), snap25 morphant (B) and snap25 MO+*snap25b* mRNA (C) embryos at 72hpf. (D,F,H) lateral views of acetylated tubulin staining showing the PLLn in control (D), snap25 morphant (F) and snap25 MO+*snap25b* mRNA (H) embryos at 4dpf. (E-I”) lateral views of sodium channels and acetylated tubulin staining along the PLLn of control (E-E”), snap25 morphant (G-G”) and snap25MO+ *snap25b* mRNA (I-I”) embryos at 4dpf. (J,K) Quantification of the sodium channel and neurofascin clustering, data are represented as mean±sem. Scale bars = 200 μm, 60 μm and 5 μm in (C), (F) and (I,I”) respectively. Transmission electron micrographs showing cross-section through (L) control and (M) snap25 morphant’s PLLn at 4 dpf. (L) Control PLLn shows an average of 10.7 myelinated axons (blue asterisks). (M) An average of 9.13 myelinated axons (blue asterisks) is also observed in the snap25 morphant embryo’s PLLn. (S: Schwann cell). Scale bars = 0.5μm. (N) Quantification of the number of nodes seen in the PLLn in controls, ndrg4 morphants and ndrg4 morphants injected with 150pg of *snap25b* mRNA. (O) Quantification of the number of nodes seen in the PLLn in controls, ndrg4 mutants and ndrg4 mutants injected with 150pg of *snap25b* mRNA.

Our analysis shows that ndrg4 can regulate the expression of several key factors involved in vesicular docking and release (S1 Table and Fig 5), including snap25 and nsfa. While it has been shown that nsf is essential for sodium channel clustering at the nodes [33], we wanted to assess whether snap25 is also involved in this process, since these two proteins are part of the t-SNARE/NSF machinery required for vesicle docking and release [27]. To test this hypothesis, we injected a specific 5’UTR morpholino against snap25a and b in zebrafish [42]. Zebrafish embryos injected with 0,6 pmoles of snap25 MO showed a significant reduction in their ability to move or to respond to a touch stimulus at 3dpf (S8 Movie). This reflects the requirement of Snap25 in synaptic vesicle transmission while no major morphological nor PLLn axonal outgrowth defects were observed (Fig 6A–6C, 6D, 6F and 6H). However, a significant reduction in the number of sodium channel and neurofascin clustering was observed along the PLLn (Fig 6E–6K). We could observe 30.94 ±2.536 sodium channel clusters in control embryos (n = 17 embryos) in comparison to 13.90 ±5.6 in snap25 moprhants (n = 20 embryos). Similar results were obtained for anti-FIGQY labeling, we could observe 30.86 ±0.54 clusters in control embryos (n = 14 embryos) in comparison to 15.78 ±1.42 clusters in snap25 morphants (n = 14 embryos). Co-injection of *snap25b* mRNA (300 pg) along with snap25 MO was able to rescue the sodium channel clustering defects (31.75 ±3.53 clusters; n = 16 embryos) and the evoked touch response test (S9 Movie), showing the specificity of this knockdown approach (Fig 6E–6J). This result strongly suggests that Snap25, like Nsf, can also regulate the clustering of sodium channels and neurofascin along the PLLn in zebrafish.

We have also analyzed axonal ensheathment in snap25 morphants using TEM. Results show no significant difference in the total number of axons (44 ±2.5 axons in snap25 morphants; n = 9 nerves from 7 different embryos vs 43.6 ±2.69 axons in controls; n = 10 nerves from 9 different embryos) nor in the number of myelinated axons in these morphants in comparison to controls (9.13 ±0.71 myelinated axons in snap25 morphants vs 10.7 ±0.68 myelinated axons in controls) (Fig 6L and 6M). This result indicates that reducing the levels of snap25 does not lead to obvious myelination defects, while it significantly decreases the clustering of sodium channels and neurofascin along the PLLn, at least at the concentration used in this study.

### Snap25 over-expression slightly but significantly enhances clustering of sodium channels in ndrg4 mutants

To test whether the decrease in the expression of Snap25 is involved in the sodium channel clustering defect observed in ndrg4 mutants, we injected snap25b mRNA (150 pg) in ndrg4 mutants and morphants. Indeed, we could observe a slight but significant increase in the number of sodium channel clustering in the injected embryos in comparison to non-injected mutants or morphants (Fig 6N and 6O), while the overexpression of Snap25 did not alter the number of sodium channel clusters in controls. We could count an average of 1.15 ±0.22 (n = 14 embryos) and 6.85 ±1.15 (n = 13 embryos) clusters in ndrg4 mutants and morphants respectively in comparison to an average of 4.35 ±0.44 (n = 13 embryos) and 15.6 ±1.1 (n = 13 embryos) clusters in snap25 mRNA injected ones. This result suggests that the decrease in Snap25 expression is partially responsible for the sodium channel clustering defect observed in these mutants and morphants.

### Tetanus toxin injected embryos do not show obvious nodes organization or peripheral myelination defects

**Fig 7:**
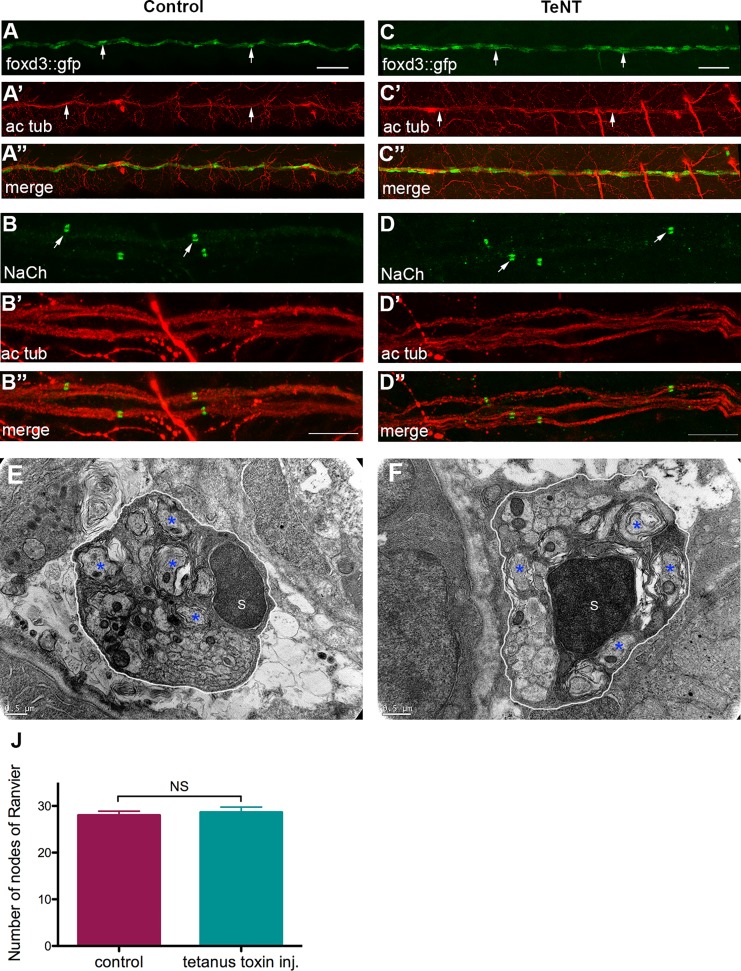
Tetanus toxin injection does not impair sodium channel clustering and myelination in the PNS. (A) Lateral view of a control foxd3::GFP embryo at 3 dpf. Arrows indicate SCs along the PLLn. (A’) Acetylated tubulin expression in the same control embryo at 3 dpf. Arrows show the PLLn axons. (A”) Merge of (A) and (A’). (C) Lateral view of a *TeNT* injected foxd3::GFP embryo at 3 dpf. Arrows indicate SCs along the PLLn. (C’) Acetylated tubulin expression in the same *TeNT* injected embryo at 3 dpf. Arrows indicate the PLLn axons. (C”) Merge of (C) and (C’). Scale bars = 50μm. Sodium channel labeling in control (B) and *TeNT* injected embryo (D) and their corresponding axons of the PLLn, (B’) and (D’) respectively. (B”) merge of (B) and (B’), (D”) merge of (D) and (D’). Scale bars = 10μm. (E,F) Transmission electron micrographs showing cross-section through (E) control and (F) *TeNT* injected embryos’ PLLn at 4 dpf. (E) Control PLLn shows an average of 6 myelinated axons (blue asterisks). (F) An average of 6 myelinated axons (blue asterisks) is also observed in the *TeNT* injected embryo’s PLLn. (S: Schwann cell). Scale bars = 0.5μm. (J) Quantification of the number of nodes seen in the PLLn shows no significant difference between controls and *TeNT* injected embryos.

Recently, it has been reported that synaptic activity can regulate myelin thickness and biases axon selection in the CNS [43,44] but it is not required *per se* for sodium channel clustering in the PNS [33]. To specifically test the role of synaptic vesicle release in myelin organization of the PLLn, we used this time the Tetanus Toxin light chain (TeNT) to investigate whether the defects observed in ndrg4 mutants are related to its role in synaptic vesicle release. Therefore, we injected *TeNT* mRNA at one cell stage so that all cells in the nervous system are affected and we analyzed the embryos at 3 and 4 dpf. This resulted, first, in a significant decrease in motility when comparing TeNT injected embryos to control ones and only embryos that showed a reduced motility were chosen for further analysis. To examine axonal integrity and SC migration, we injected *TeNT* in the foxd3::GFP line and then performed an acetylated tubulin staining at 3 dpf. We did not observe any obvious difference in axonal integrity or SC development and distribution between *TeNT* injected embryos (n = 14 embryos) (Fig 7C–7C”) and controls (n = 24 embryos) (Fig 7A–7A”). We then looked for sodium channel clustering in *TetNT* injected embryos, and we observed no difference in the number or organization of these channels along the axons in the injected embryos (average of 28.7 nodes from 21 embryos) in comparison to controls (average of 25.5 nodes from 12 embryos) (Fig 7B–7B”, 7D–7D” and 7J). We then checked the nerve ultrastructure by TEM at 4 dpf (Fig 7E and 7F) and we counted an average number of 6 myelinated axons per nerve in *TeNT* embryos (4 nerves from 3 different larvae) (Fig 7E) and in control embryos (4 nerves from 4 different larvae) (Fig 7F).

These results show that TeNT injected embryos do not show obvious sodium channel clustering and early myelin compaction defects in the PLLn.

## Discussion

The identification of ndrg4 in a differential screen looking for dysregulated genes in the absence of SCs in zebrafish, led us to generate and analyze the ndrg4 mutant. We show that ndrg4 is a novel factor required within neurons for the clustering of sodium channels along the peripheral axons. Ndrg4 mutants have severe heart defects, consistent with the potential role of *NDRG4* in heart development and function previously reported in humans. However, *Ndrg4* mice knockout show no sign of heart dysfunction [21,23,25,45–47], suggesting that the zebrafish ndrg4 mutant is a more suitable model to study the role of ndrg4 in this process. The ndrg4 mutants also show little or no mobility, most likely due to the sharp decrease in the expression of key genes involved in vesicle fusion and release therefore inhibiting synaptic neurotransmission and activity. Labeling of SV2 vesicles in ndrg4 mutants show clearly that these vesicles are present along the axons but tend to agglomerate, suggesting a defect in their release. Reducing the levels of Snap25 in zebrafish embryos leads to a significant decrease in the clustering of sodium channels and over-expression of Snap25 in ndrg4 mutants slightly but significantly increases the clustering of sodium channels along the PLLn. Moreover, monitoring vesicle transport along these axons shows no obvious abnormality in comparison to controls. Overall, these results point to a rather global vesicle docking and release defect along the ndrg4 mutant axons rather than to an abnormal vesicle formation and transport. Moreover, they indicate that these processes can be dissociated. The reduction in the number of myelinated axons observed in ndrg4 mutants and morphants’ PLLn suggests that its function is not required *per se* for SC radial sorting or myelination. The myelin defect might be the result of a delay in the development of these embryos given their severe heart defects or a defective axonal-SCs interaction that alters SC myelination process. However, ndrg4 mutants do not survive beyond 5–6 dpf to test this hypothesis. Overall, this establishes ndrg4 as a novel member of the Ndrg family, to add to NDRG1, that plays a fundamental role in the organization of myelinated axons in the PNS.

### A novel neuronal ndrg4 function that regulates sodium channel clustering

We here identify a novel neuronal function for zebrafish ndrg4 required for sodium channel and neurofascin clustering along the PLLn. Ndrg4 regulates, among others, the expression of several key genes involved in vesicle fusion and release [26,27]. It has been shown that ion channels, particularly Nav1.2 channels, require vesicular axonal transport from the neuronal cell body to be later anchored at the sites of the nodes [40,41]. This process is mediated by ankyrin-G and kinesin-1, however less is known about other fundamental players required for ion channel docking. One particular mutant that shows comparable myelinated axons organization defects to ndrg4 is the *nsf* mutant. Neuronal nsf is autonomously required for sodium channel clustering in the PLLn [33,48] and is characterized as an essential component for vesicle fusion and release by interacting with and dissociating the SNARE complex [27,49,50]. Moreover, other data show nsf requirement for Ca^2+^ channels localization and function in nerve terminals [51].

Overall, based on previous studies and our data presented here, it is now clear that i) both nsf and ndrg4 mutants cause a very severe defect in sodium channel and neurofascin clustering along the axons, ii) ndrg4 loss leads to a sharp decrease in Snap25 and nsf expression, iii) snap25 knockdown leads to a significant decrease in sodium channel clustering, iiii) snap25 over-expression slightly but significantly enhances sodium channel clustering in ndrg4 mutants and iiiii) both NSF and SNAP25 have a fundamental role, *in vivo*, in vesicular docking and release. We thus propose that key components of the NSF/t-SNARE machinery, tightly controlled by ndrg4, are most likely playing an essential role in sodium channel and neurofascin clustering in the PNS, independent of their role in synaptic vesicle release. It has been shown that axonal adhesion molecules e.g. neurofascin are diffusible and cluster at the nodes from adjacent axonal domain while sodium channel clustering relies on vesicular axonal transport in the PNS [41]. Our data show that sodium channel and neurofascin clustering are both defective in ndrg4 mutants and snap25 morphants, and similar results were obtained with nsf mutant [33]. Given that axonal vesicular transport is not dependent on ndrg4 function but vesicle docking is, one possibility is that the initial anchoring of neurofascin and sodium channels along the axons might rely on vesicle docking. It would be interesting to test whether these fundamental components of the t-SNARE/NSF machinery are also involved in sodium channel and neurofascin clustering at the nodes in mice, and to carefully analyze the clustering of sodium channels and neurofascin in Ndrg4 KO mice. Ndrg4-/- mice exhibits inferior performance in escape latency and total path lengths in the MWM task in comparison to controls but this is not comparable to the total lack of mobility seen in zebrafish ndrg4 mutants. Moreover, Ndrg4-/- mice do not show any heart defects [23] in contrast to zebrafish mutants. Whether ndrg4 function in the heart and nodes assembly is specific to zebrafish or a possible redundancy would explain the lack of defects in Ndrg4 KO mice is to be tested in the future.

### Ndrg4 in vesicle fusion and synaptic activity

Several lines of evidence presented here suggest a potential role of ndrg4 in controlling synaptic vesicle release *in vivo* (S1 Table). Numerous studies indicated a role of synaptic activity in myelination and nodes of Ranvier establishment but conflicting results emerged [52,53]. However, the first *in vivo* evidence, using zebrafish, showed no significant requirement of synaptic activity in PNS sodium channel clustering and *mbp* expression using tetrodotoxin (TTX) and neomycin [33]. We here injected TeNT at one cell stage so that the whole nervous system is affected and whereby the Ca^2+^ triggered exocytosis is specifically down-regulated while the constitutive one is not impaired [54]. We show using TEM that the PNS myelin is comparable to controls and that nodes organization is also similar to controls suggesting that synaptic vesicle release is not required, *per se*, for PNS myelin organization. However, whether these drugs have the same effect on synaptic vesicle release in the PNS, as shown in the CNS [44,55], should be carefully tested in the future. Synaptic vesicle release might be responsible for myelin ensheathment in the CNS as it has been proposed a synaptic-like interaction between OLs and axons [43,44,56], nevertheless its role in nodes organization has not been tested yet. SNAP25 and NSF have been shown to be involved in both types of constitutive and regulated exocytosis as SNARE proteins and NSF are essential for all intracellular membrane fusion events [26,27,57]. We here show that Snap25 expression is decreased within neurons and along the axons of the PLLg in the ndrg4 mutants and morphants, suggesting a role of ndrg4 in controlling both regulated and constitutive vesicle release. However, a defect in the latter is more likely to be responsible for nodes disorganization in these mutants. A recent study shows a role of ndrg4 in exocytosis by regulating Fibronectin recycling and secretion via its interaction with the Blood Vessel Epicardial Substance (Bves) to control epicardial cell movement [58]. Ndrg4 is mainly expressed in the nervous system and heart showing a rather specific temporal and local control of snap5 and nsf expression by ndrg4 during nervous system development. Indeed, the mRNA expression of ndrg4, snap25 and nsf are identical at 48 and 72 hpf, (at least in the PLLg; S4 Fig and [33]).

Overall, these data reveal an unknown neuronal function of ndrg4 in vesicle release and peripheral myelinated axons organization that is most likely due to its role in controlling the expression of key components of the t-SNARE/NSF machinery.

## Materials and Methods

### Embryo care

Embryos were staged and cared for according to standard protocols. Foxd3::GFP [37], Sox10::mRFP [29] and HuC::GFP [59] stable transgenic lines, that label SCs and neurons, some of which previously described in [60] were used in this study. All animal experiments were conducted with approved protocols at Inserm.

### ndrg4 CRISPR mutagenesis

#### sgRNA generation

sgRNA guide sequence (GGTGATCCGTGGCGCTCCCA), targeting *ndrg4* exon 2, was cloned into the DR274 (Addgene 42250) vector digested with BsaI. *In vitro* transcription of the sgRNA was performed using the Megascript T7 transcription kit (Ambion AM1334) and sgRNA was purified using RNeasy Mini Kit (Qiagen).

#### Injections

To induce targeted mutagenesis at the *ndrg4* locus, 200 ng/ul of sgRNA were injected into one-cell stage zebrafish embryos together with Cas9 endonuclease (NEB M0386M; final concentration: 25 μM). Pools of embryos were digested to extract genomic DNA (to perform PCR and DNA sequencing experiments).

Injected embryos were grown to adulthood and screened for mutation in their offspring. Two different mutants that showed a deletion of 10 and 11 nucleotides respectively were used in this study (See Fig 1).

#### Whole embryos DNA extraction and mutation analysis

Embryos were digested for 1 h at 55°C in 0.5mL lysis buffer (10 mM Tris, pH 8.0, 10 mM NaCl, 10 mM EDTA, and 2% SDS) with proteinase K (0.17 mg/mL, Roche Diagnostics) to extract genomic DNA. To verify cleavage at targeted sequence, ndrg4 exon 2 was PCR amplified and digested with the restriction enzyme HaeII. The restriction site, placed in the sgRNA-binding region, would be removed upon Indel mutations at the *ndrg4* locus. To estimate the rate of mutations *ndgr4* amplicons were cloned into the pCR-bluntII-TOPO vector (life technologies 450245). Single amplicons were sent for sequencing and mutant alleles were identified by comparison to the wild-type unmodified sequence. Primers used for the PCR were: ndrg4 fw: 5’-CCTGCAAACAAGCAAGCCA-3’ and ndrg4 rev: 5’-ATCATCCTCGTCTCACGCTG-3’.

### Microinjections

Splice blocking *ndrg4-*MO (5’-TGCATTCATCTTACCCTTGAGGCAT-3’), 5mis *ndrg4-*MO (5’-TGgATTgATCTTAgCCTTcAGGgAT-3’), described in (21), and 5’UTR *snap25-*MO (5’-AGCTGCTCTCCAACTGGCTCTTACT-3’) described in (42) were purchased from Gene Tools.

We used a corresponding ndrg4 5-mis MO as a control in all our experiments. There were no significant difference between control injected embryos and Wild Type (WT) ones. For convenience, we refer to control as WT, non ndrg4-/- mutants and 5-mis MO injected embryos in the Figures, unless it is stated otherwise.

For ndrg4 rescue experiment, *ndrg4* mRNA was synthesized using SP6 mMessage mMachine System after linearization with Not1. For snap25 rescue and overexpression experiments, *snap25b* mRNA was synthetized using T3 mMessage mMachine System after linearization with Apa1.

For TeNT experiments, tetanus toxin light chain cDNA was purchased from Addgene. Synthetic *TeNT* mRNA was generated using SP6 mMessage mMachine System after linearization with SacII and injected at 150 pg per embryo. Rab5::YFP (a gift from Carl-Philipp Heisenberg) and mito::GFP (a gift from Dominik Paquet) mRNAs were synthesized using SP6 mMessage mMachine System after linearization with Not1 and injected at 200 pg per embryo.

### In situ hybridization

*In situ* hybridization was performed following standard protocols previously described in [60] using *mbp* [48] and *sox10* probes [61]. ndrg4, and snap25b cDNA clones were purchased from Source BioScience UK. *ndrg4*, *snap25b* antisense probes were synthesized using mMessage mMachine System (Ambion) and T7 polymerase after linearization with EcoR1 for ndrg4 and NotI for snap25b.

### Microarray hybridization

RNA was extracted from two groups of zebrafish embryos (1.control embryos and 2. ndrg4 morphants) at 3dpf, cDNA generated and applied to Zebrafish_v3 4x44K array (Agilent Technologies). Significantly different genes were first selected using *GeneSpring* 12.0 (Agilent Technologies) and then filtered using t-test and genes with a p value of less than 0.05 were filtered out.

### Quantitative real-time RT-PCR

RNA was extracted using Trizol reagent (Life Technologies) and miRNeasy Mini kit (Qiagen) according to manufacturer’s instructions. For mRNA quantitation, Reverse Transcription (RT) was performed using High Capacity cDNA Reverse Transcription Kit (Life Technologies). Quantitative real-time PCR (qPCR) was performed using Power SYBR-Green Master Mix (Biorad) on an Applied 7500 Real-Time PCR system. Primers used for qPCR are listed here:

Nsf1a,

forward: CGCGGCTTCTTCGAGTAACA

reverse: GAAGTGTGATCTCCGTCAGGTT

Syt1a,

Forward: AAAGGGAAGAGACGGCTGTG

Reverse: GGAGCCAGGCAGAAGCTTTA

Stxbp1b,

Forward: ACGCTGAAAGAGTACCCAGC

Reverse: CTCCCAAAGTGGGGTCATCC

Vamp2,

Forward: CGCAACATTCCTACCCCACT

Reverse: GTGAGAAGTCGTTGCTCCCA

mRNA expression levels in wild type or ndrg4 mutant zebrafish were determined by RT-qPCR. mRNA amount was normalized to that of EF1-a mRNA then expressed as a relative amount to WT (data represent the mean ± SD of triplicates).

### Immunofluorescence

The following antibodies and dilutions were used: mouse anti-acetylated tubulin (Sigma; 1:500), rabbit anti-PH3 (Millipore; 1:500), mouse anti-SNAP25 (Synaptic Systems; 1:200), mouse anti-sodium channels (pan) clone K58/35 (Sigma; 1:500), mouse anti-SV2 (DSHB; 1:200), rabbit anti FIGQY (a gift from Matthew Rasband; 1:500). Primary antibodies were detected with appropriate secondary antibodies conjugated to either Alexa 488 or Alexa 568 (Molecular probes) at a 1:1000 dilution.

For immunostaining, embryos were fixed in 4% paraformaldehyde 1X PBS overnight at 4°C and stained as whole mounts. Sodium channels, SNAP25 and SV2 immunostainings were performed as described for Na_v_Ch staining in [33].

Images were taken on a Zeiss LSM510 system and a Leica SP8 confocal microscope.

### Live imaging

Embryos were anesthetized with tricaine and embedded in 1.5% low melting point agarose. For mito::GFP and rab5::YFP tracking experiments, PLLn was examined at 48 hpf from a lateral view. A series of 10 minutes time-lapses were recorded. Recordings were performed at 28°C using a Leica SP8 confocal microscope. Larval movements stimulated by touch-response test were performed at room temperature and recorded using a Zeiss Lumar.V12 stereoscope and Zeiss AxioCam MRc camera.

### Western blot

Proteins were extracted from pools of embryos as previously described in [62] with 10μl lysis buffer (1M Tris HCl pH 6.8, glycerol 40% and SDS 10%) per embryo. Protein content was determined using the Pierce BCA protein assay. 25 μg proteins were loaded on gel. Western blots were performed according to standard methods using the following antibodies: mouse anti-snap25 (Synaptic Systems; 1:1000), mouse anti-β-actin (Sigma, clone AC-15; 1:10,000) and appropriate HRP-conjugated secondary antibodies (Jackson immuno research).

### Transmission electron microscopy

At 4 dpf, embryos were fixed in a solution of 2% glutaraldehyde, 2% paraformaldehyde and 0.1M sodium cacodylate pH 7.3 overnight at 4°C. This was followed by a post-fixation step in cacodylate-buffered 1% osmium tetraoxide (OsO_4_, Serva) for 1h at 4°C and in 2% uranyl acetate for 1h at room temperature. The tissue was then dehydrated and embedded in epoxy resin. Sections were contrasted with saturated uranyl acetate solution and were examined with a 1011 electron microscope (JEOL) and a digital camera (Gatan).

### Chimeric analysis

Donor cells were injected with 0.6pmoles of ndrg4MO and mCherry mRNA (300ng/μl) or with mCherry mRNA (300ng/μl) and introduced into a WT background. mCherry WT cells were also introduced into ndrg4 morphant background. In all cases, only embryos that presented labeling in the nervous system were further analyzed for sodium channel clustering.

### Statistical analysis

Means and standard deviations were calculated with Microsoft Excel version 14.4.3 or Graph Pad Prism 5. Means were compared by the two-tailed Student’s *t* test or one-way ANOVA according to the experiment. p<0.05 was considered statistically significant.

### Ethics statement

All experiments were carried out in accordance to the official regulatory standards of the Department of Val de Marne (agreement number D 94-043-013 to the animal facility of Bâtiment Pincus, Institut Biomédical de Bicêtre).

## Supporting Information

S1 Fig ndrg4 morphant and mutant phenotype. (A-C) Overall morphology of a control embryo (A), *ndrg4* morphant embryo (B), ndrg4 MO+*ndrg4* mRNA co-injected embryo (C), at 48hpf. (B) ndrg4 morphant embryo displays smaller head and eyes and is slightly thinner. (C) Co-injection of *ndrg4* MO and mRNA rescue this phenotype. Scale bars = 500μm. (D-F) Acetylated tubulin and sodium channels staining of a *ndrg4* MO+mRNA co-injected embryo showing clustered sodium channels along the PLLn (34.2 ±3.2; n = 12 embryos) similar to controls. Scale bar = 5μm. (G) Sodium channels staining in sox10::mRFP transgenic line in controls and ndrg4 morphants. Arrowheads indicate the clustering of sodium channels while arrows point to nodal gaps. Note the absence of sodium channels clustering at the nodes in ndrg4 morphants. Scale bar = 5 μm. (H-J) Snap25 immunostaining in HuC::GFP larvae at 3dpf. (H) Control embryos show Snap25 expression in PLLg neurons (arrows). (I) A reduced Snap25 expression was observed within the PLLg in ndrg4 morphants. (J) Rescue of Snap25 expression in the PLLg in ndrg4MO+mRNA co-injected embryos. Scale bars = 5μm. (K) Snap25 expression in the PLLg of control and ndrg4-/- embryos. Note the significant decrease in the expression of Snap25 within the PLLg of ndrg4-/- in comparison to controls.
(TIF)

S2 Fig ndrg4 is not required for SC proliferation. PH3 immunohistochemistry in (A) control and (B) ndrg4 morphant foxd3::GFP embryos at 48 hpf. Arrows indicate dividing PLLn SCs. Scale bar = 100μm. (C) Quantification of PH3 positive SCs in controls and ndrg4 morphants shows no significant differences between the two groups at 30 hpf, 48 hpf and 72 hpf.
(TIF)

S3 Fig *ndrg4* mRNA is expressed in the PLL ganglion and not in SCs. (A-C) *In situ* hybridization showing *ndrg4* mRNA expression in the brain, eye and in the PLL ganglion (arrow) at 30 hpf (A), 48 hpf (B) and 72 hpf (C). Scale bar = 200μm.
(TIF)

S4 Fig *Snap25* mRNA is expressed in the PLL ganglion. *In situ* hybridization showing *snap25b* mRNA expression in the brain, eye and in the PLL ganglion (arrow) at 48 hpf and 72 hpf. Scale bar = 200μm.
(TIF)

S1 Movie Touch response mobility of 3dpf WT larvae. (MP4)

S2 Movie The ndrg4 mutants are paralyzed and do not respond to a touch-evoked motility test at 3dpf. (MP4)

S3 Movie The ndrg4 morphant is paralyzed and shows no response following touch-evoked motility test at 48hpf. (MP4)

S4 Movie Real-time imaging of mitochondria in a control PLLn at 48 hpf. Forty-eight hours embryo expressing GFP in mitochondria after *mito*::*GFP* mRNA injection; the embryo was imaged every 4 seconds for 6 minutes by confocal microscopy. Lateral view; anterior to the left and dorsal to the top.
(MOV)

S5 Movie Real-time imaging of mitochondria in a ndrg4 morphant PLLn at 48 hpf. Forty-eight hours ndrg4 morphant expressing GFP in mitochondria after *mito*::*GFP* mRNA injection; the embryo was imaged every 4 seconds for 6 minutes by confocal microscopy. Lateral view; anterior to the left and dorsal to the top.
(MOV)

S6 Movie Real-time imaging of Rab5 positive vesicles in an ndrg4 morphant PLLn at 48 hpf. Forty-eight hours ndrg4 morphant expressing YFP in early endosomes after *Rab5*::*YFP* mRNA injection; the embryo was imaged every 425 milliseconds for 2 minutes by confocal microscopy. Lateral view; anterior to the left and dorsal to the top.
(MOV)

S7 Movie Real-time imaging of Rab5 positive vesicles in a control PLLn at 48 hpf. Forty-eight hours embryo expressing YFP in early endosomes after *Rab5*::*YFP* mRNA injection; the embryo was imaged every 425 milliseconds for 2 minutes by confocal microscopy. Lateral view; anterior to the left and dorsal to the top.
(MOV)

S8 Movie The snap25 morphants show little or no movement. (MP4)

S9 Movie Co-injection of snap25 MO and *snap25b* mRNA restores defective evoked touch response observed in snap25 morphants at 3dpf. (MP4)

S1 Table A selection of genes involved in vesicular docking and release or synaptic activity that are down regulated in ndrg4 morphants in comparison to controls at 3dpf. Genes are sorted in a descending order related to their fold change.
(DOC)
