# Supplementary figures and images for: Neuronal Ndrg4 Is Essential for Nodes of Ranvier Organization in Zebrafish
Source: PLoS Genet. 2016 Nov 30;12(11):e1006459. doi: 10.1371/journal.pgen.1006459 (PMC5130175; doi:10.1371/journal.pgen.1006459)

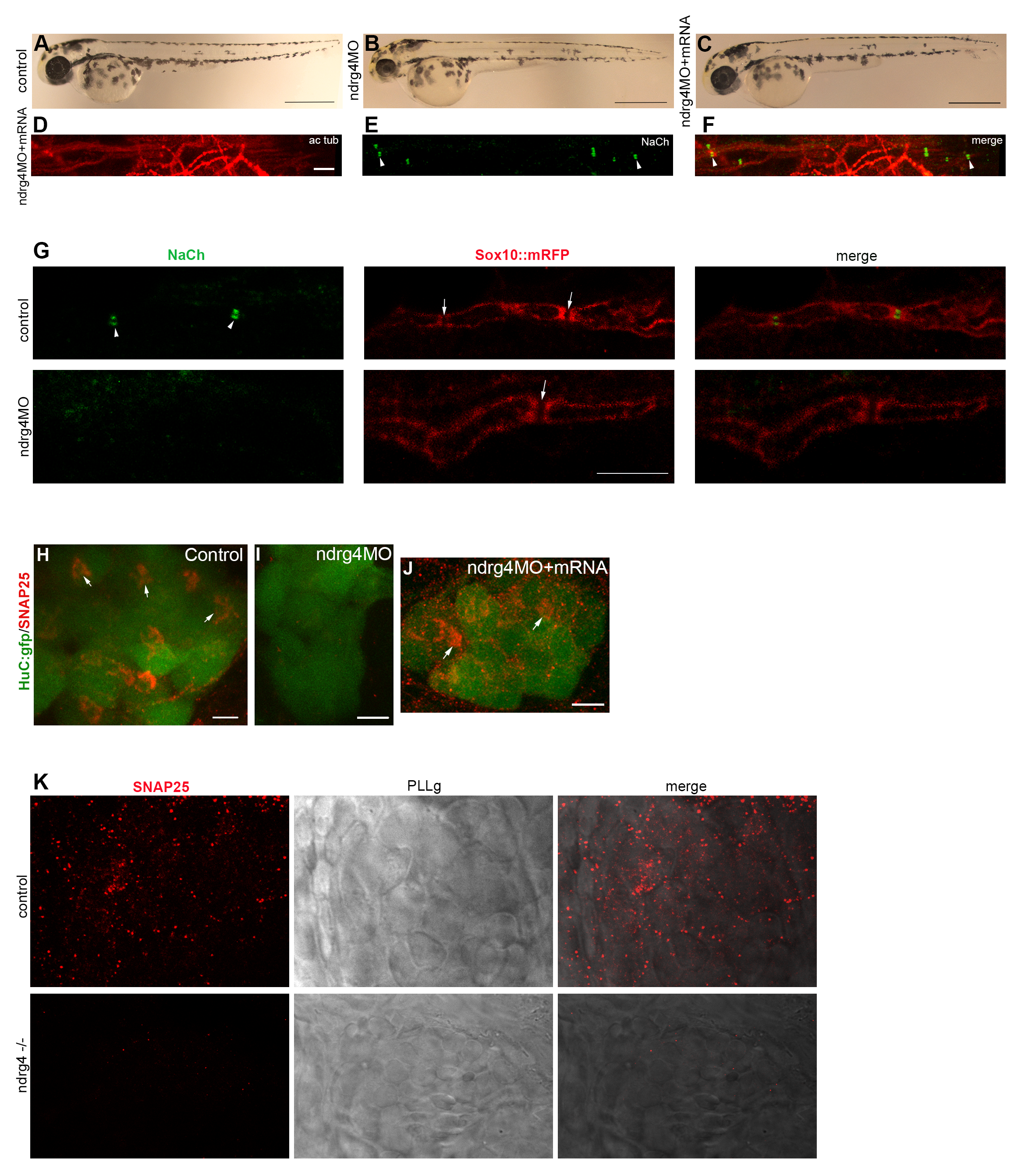

Supplement: S1 Fig — (A-C) Overall morphology of a control embryo (A), ndrg4 morphant embryo (B), ndrg4 MO+ndrg4 mRNA co-injected embryo (C), at 48hpf. (B) ndrg4 morphant embryo displays smaller head and eyes and is slightly thinner. (C) Co-injection of ndrg4 MO and mRNA rescue this phenotype. Scale bars = 500μm. (D-F) Acetylated tubulin and sodium channels staining of a ndrg4 MO+mRNA co-injected embryo showing clustered sodium channels along the PLLn (34.2 ±3.2; n = 12 embryos) similar to controls. Scale bar = 5μm. (G) Sodium channels staining in sox10::mRFP transgenic line in controls and ndrg4 morphants. Arrowheads indicate the clustering of sodium channels while arrows point to nodal gaps. Note the absence of sodium channels clustering at the nodes in ndrg4 morphants. Scale bar = 5 μm. (H-J) Snap25 immunostaining in HuC::GFP larvae at 3dpf. (H) Control embryos show Snap25 expression in PLLg neurons (arrows). (I) A reduced Snap25 expression was observed within the PLLg in ndrg4 morphants. (J) Rescue of Snap25 expression in the PLLg in ndrg4MO+mRNA co-injected embryos. Scale bars = 5μm. (K) Snap25 expression in the PLLg of control and ndrg4-/- embryos. Note the significant decrease in the expression of Snap25 within the PLLg of ndrg4-/- in comparison to controls. (TIF) [file pgen.1006459.s001.tif]

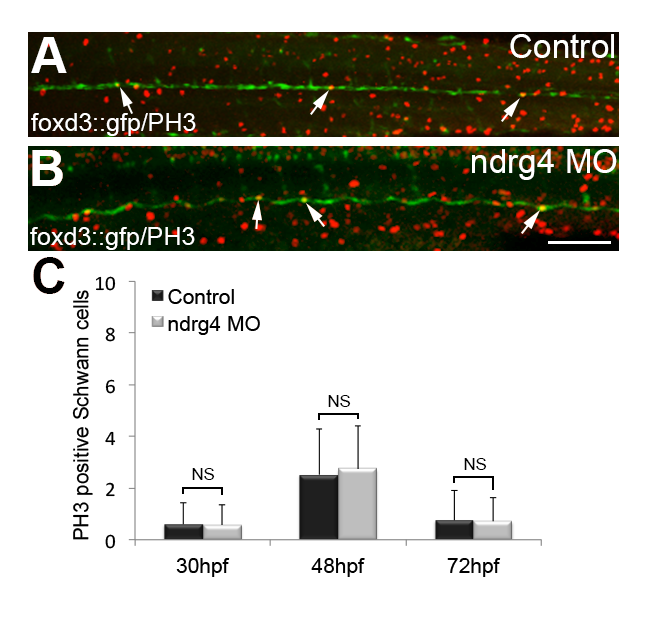

Supplement: S2 Fig — PH3 immunohistochemistry in (A) control and (B) ndrg4 morphant foxd3::GFP embryos at 48 hpf. Arrows indicate dividing PLLn SCs. Scale bar = 100μm. (C) Quantification of PH3 positive SCs in controls and ndrg4 morphants shows no significant differences between the two groups at 30 hpf, 48 hpf and 72 hpf. (TIF) [file pgen.1006459.s002.tif]

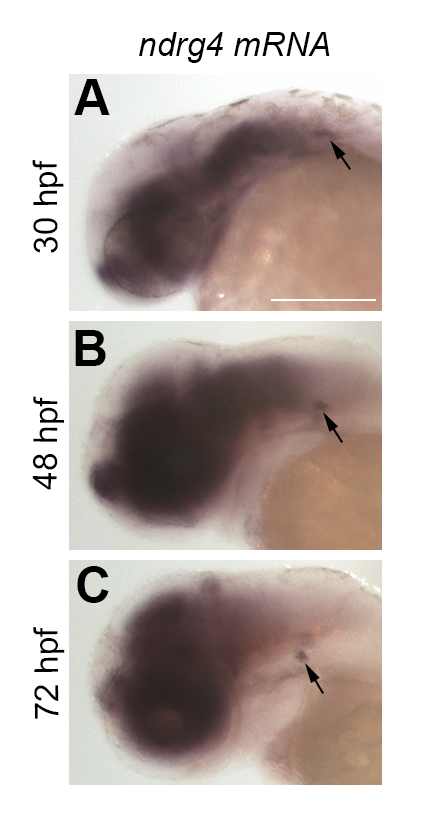

Supplement: S3 Fig — (A-C) In situ hybridization showing ndrg4 mRNA expression in the brain, eye and in the PLL ganglion (arrow) at 30 hpf (A), 48 hpf (B) and 72 hpf (C). Scale bar = 200μm. (TIF) [file pgen.1006459.s003.tif]

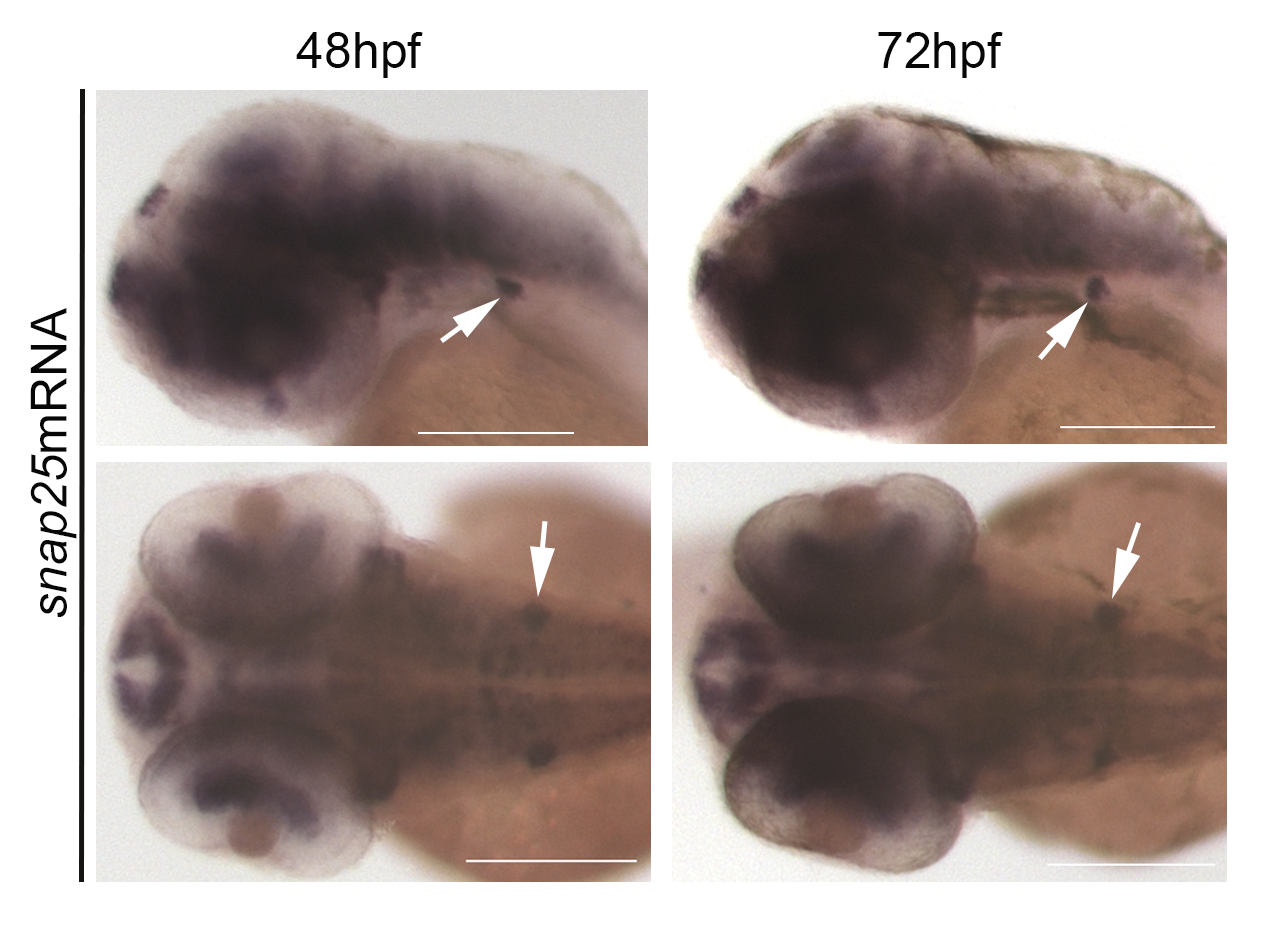

Supplement: S4 Fig — In situ hybridization showing snap25b mRNA expression in the brain, eye and in the PLL ganglion (arrow) at 48 hpf and 72 hpf. Scale bar = 200μm. (TIF) [file pgen.1006459.s004.tif]
