# Supplementary material for: Neuronal Ndrg4 Is Essential for Nodes of Ranvier Organization in Zebrafish
Source: PLoS Genet. 2016 Nov 30;12(11):e1006459. doi: 10.1371/journal.pgen.1006459 (PMC5130175; doi:10.1371/journal.pgen.1006459)
Supplement: S1 Table — Genes are sorted in a descending order related to their fold change. (DOC) [file pgen.1006459.s014.doc]

S1 Table

| **Gene Symbol** | **Fold change** | **Gene description** |
| --- | --- | --- |
| CALY | 29 | calcyon neuron-specific vesicular protein |
| SLC1A7B | 10 | solute carrier family 1 (glutamate transporter), member 7b |
| CNG4 | 9.9 | cyclic-nucleotide-gated cation channel 4 |
| STXBP1A | 6.3 | syntaxin binding protein 1a |
| SLC1A6 | 4.3 | solute carrier family 1 (high affinity aspartate/glutamate transporter), member 6 |
| SNAP25B | 3 | synaptosomal-associated protein, 25b |
| GABRA1 | 2.97 | gamma-aminobutyric acid (GABA) A receptor, alpha 1 |
| STXBP1B | 2.95 | syntaxin binding protein 1b |
| NRXN1L | 2.94 | neurexin 1 like |
| SYN2B | 2.86 | synapsin IIb |
| SLC17A7A | 2.85 | solute carrier family 17 (vesicular glutamate transporter), member 7a |
| SYN2A | 2.59 | synapsin IIa |
| RAB3B | 2.50 | RAB3B, member RAS oncogene family |
| SYT1A | 2.43 | synaptotagmin Ia |
| NSFA | 2.40 | N-ethylmaleimide-sensitive factor a |
| VAMP1 | 2.40 | vesicle-associated membrane protein 1 |
| SYPA | 2.19 | synaptophysin a |
| RIMS2A | 2.09 | regulating synaptic membrane exocytosis 2a |
| ERC1A | 2.04 | ELKS/RAB6-interacting/CAST family member 1a |
| SYNGR3 | 1.99 | synaptogyrin 3 |
| SNIP | 1.87 | p130Cas-associated protein (p140Cap) (SNAP-25-interacting protein) |
